# Prediagnostic circulating levels of sex hormones and survival in esophageal adenocarcinoma

**DOI:** 10.1002/ijc.33285

**Published:** 2020-09-22

**Authors:** Shao‐Hua Xie, Eivind Ness‐Jensen, Hilde Langseth, Randi E. Gislefoss, Fredrik Mattsson, Jesper Lagergren

**Affiliations:** ^1^ Upper Gastrointestinal Surgery, Department of Molecular medicine and Surgery Karolinska Institutet, Karolinska University Hospital Stockholm Sweden; ^2^ HUNT Research Centre, Department of Public Health and Nursing NTNU, Norwegian University of Science and Technology Levanger Norway; ^3^ Medical Department, Levanger Hospital Nord‐Trøndelag Hospital Trust Levanger Norway; ^4^ Cancer Registry of Norway Institute of Population‐Based Cancer Research, Department of Research Oslo Norway; ^5^ Department of Epidemiology and Biostatistics Imperial College London London UK; ^6^ School of Cancer and Pharmaceutical Sciences King's College London London UK

**Keywords:** adenocarcinoma, esophageal neoplasms, gonadal steroid hormones, mortality, prognosis

## Abstract

Sex hormonal differences may contribute to the strong male predominance in esophageal adenocarcinoma (EAC), but whether sex hormone levels influence survival in EAC is unstudied. Our study aimed to assess associations between prediagnostic sex hormone levels and survival in EAC. In a population‐based cohort study, 244 male EAC patients from the Janus Serum Bank Cohort in Norway were followed up through 2018. Associations between prediagnostic serum levels of 12 sex hormone measures and disease‐specific mortality were assessed using multivariable Cox regression, providing hazard ratios (HR) with 95% confidence intervals (CI) adjusted for age, calendar year, body mass index, tobacco smoking, physical activity and surgical resection. Higher levels of sex hormone‐binding globulin (SHBG) indicated decreased disease‐specific mortality (HR 0.68, 95% CI 0.44‐1.07, highest vs lowest tertile). In stratified analyses by surgery, such associations remained in nonoperated patients (HR 0.58, 95% CI 0.35‐0.96, highest vs lowest tertile), but not in operated patients. Higher levels of follicle‐stimulating hormone (FSH) were associated with increased disease‐specific mortality in an exposure‐response pattern; HRs for the middle and highest tertiles vs the lowest tertile were 1.35 (95% CI 0.89‐2.05) and 1.61 (95% CI 1.06‐2.43), respectively. No clear associations were observed with serum levels of dehydroepiandrosterone sulfate, luteinizing hormone, prolactin, testosterone, 17‐OH‐progesterone, progesterone, estradiol, androstenedione, testosterone:estradiol ratio or free testosterone index. These findings suggest that higher endogenous levels of SHBG and lower levels of FSH may increase the survival in EAC. The other 10 examined sex hormone measures may not influence the survival.

## Abstract 2

**What's new?**

Esophageal adenocarcinoma (EAC) occurs more frequently in men than women. Whether this pattern is linked to differences in sex hormone levels and whether such differences impact EAC survival remain unclear. In this study of male EAC patients in Norway, analyses of prediagnostic sex hormone measures uncovered associations between reduced disease‐specific mortality and increased sex hormone‐binding globulin and decreased follicle‐stimulating hormone levels. The associations were detected only in patients who had not undergone surgery. Ten other sex hormone measures also analyzed had no influence on survival. Additional investigation is needed to better understand relationships between sex hormone levels and EAC survival.

**Abbreviations**
BMI: body mass index
CI: confidence interval
EAC: esophageal adenocarcinoma
FSH: follicle‐stimulating hormone
HR: hazard ratio
ICD: International Classification of Diseases
SHBG: sex hormone‐binding globulin

## 1 INTRODUCTION

The incidence of esophageal adenocarcinoma (EAC) has increased rapidly during the past four decades, and EAC has become the predominant histologic type of esophageal malignancy in many Western countries.^1^ The overall 5‐year survival rate in EAC is below 20%, with tumor stage at diagnosis as the strongest prognostic factor.^1^, ^2^, ^3^, ^4^ Surgical resection (esophagectomy) alone or combined with chemotherapy or chemoradiotherapy is the main curative treatment in most patients.^2^

EAC is overrepresented in men, with a male‐to‐female incidence ratio of up to 9:1.^5^, ^6^ This striking male predominance may be explained by the differences in endogenous sex hormonal levels between the sexes.^1^, ^6^ Recent studies have reported associations between circulating sex hormone levels and the risk of developing EAC and its precursor Barrett's esophagus.^7^, ^8^, ^9^, ^10^, ^11^ Any procarcinogenic or anticarcinogenic effects of certain sex hormones could potentially influence treatment efficacy and risk of tumor recurrence after treatment of EAC. Studies have found expression of sex hormone receptors in EAC and correlations between the status of estrogen receptor beta and more advanced tumor stage at EAC diagnosis.^12^, ^13^ Yet, no study has thus far examined whether endogenous sex hormone levels influence the survival in EAC patients.

Our study aimed to assess associations between prediagnostic circulating levels of sex hormones and survival in EAC patients.

## 2 METHODS

### 2.1 Study design and participants

This prospective cohort study consisted of 244 newly diagnosed male EAC patients, who were identified from the population‐based Janus Serum Bank Cohort.^14^ The present study was based on five health surveys in different counties of Norway conducted in the 1970s to 1990s, with 293 000 participants who accounted for about 90% of participants in the Janus Serum Bank Cohort.^15^ Linkage to the Cancer Registry of Norway enabled identification of all new male EAC patients during the follow‐up of the cohort until 31 December 2016. EAC was defined by a combination of the diagnosis code for esophageal cancer (C15) in the International Classification of Diseases, 10th version (ICD‐10), and the histological codes for adenocarcinoma (8140‐8141, 8143‐8145, 8190‐8231, 8260‐8263, 8310, 8401, 8480‐8490, 8550‐8551, 8570‐8574 or 8576) in the International Classification of Diseases for Oncology, Third Edition (ICD‐O‐3).^16^ We did not include female patients because of the low incidence of EAC and the great menstrual variations in sex hormone levels in women. The male EAC patients were followed up from the date of diagnosis until death, emigration or the end of the study (31 December 2018), whichever occurred first. Date and causes of death and emigration were identified through linkage to the Norwegian Cause of Death Registry. The 11‐digit unique personal identification numbers assigned to all Norwegian residents enabled the linkages of individual data from participants in the Janus Serum Bank Cohort to the Cancer Registry and Cause of Death Registry. The completeness of both these registries is close to 100%.^17^, ^18^

### 2.2 Hormone level exposures

The participants donated serum samples when they entered the Janus Serum Bank Cohort. To assess the validity of the archived samples, repeated stability experiments have confirmed that relevant serum components are stable after long‐term storage.^19^, ^20^, ^21^, ^22^ We evaluated the following 12 sex hormone measures in serum samples: sex hormone‐binding globulin (SHBG); the nine steroid sex hormones dehydroepiandrosterone sulfate, follicle‐stimulating hormone (FSH), luteinizing hormone, prolactin, testosterone, 17‐OH‐progesterone, progesterone, estradiol and androstenedione; and finally calculated free testosterone index (testosterone × 10/SHBG) and testosterone:estradiol ratio. These hormone measures cover key points in the biosynthesis of sex hormones,^7^, ^11^, ^23^ and are at detectable levels in serum in men with the available analytic methods. These analyses were conducted at the Hormone Laboratory, Oslo University Hospital. Testosterone, 17‐OH‐progesterone and androstenedione were analyzed using liquid chromatography‐mass spectrometry, and the remaining hormones were analyzed using immunoassays, all according to standard laboratory protocols.^24^

### 2.3 Mortality outcomes

The main outcome was disease‐specific mortality after diagnosis of EAC, defined by esophageal cancer as the underlying cause of death, through 31 December 2017 (the latest date for which data on causes of death were available). The secondary outcome was all‐cause mortality after EAC diagnosis until end of the entire study period, that is, 31 December 2018.

### 2.4 Covariates

The covariates considered were six factors that might influence the survival in EAC: age, calendar year at diagnosis, body mass index (BMI), tobacco smoking, physical activity and curatively intended esophagectomy. Data on age, calendar year and surgery were retrieved from the Cancer Registry of Norway, whereas information about BMI, smoking and physical activity came from questionnaires completed at baseline, that is, when the participants were recruited to the Janus Serum Bank Cohort. The assessment of the questionnaire data has been described in detail elsewhere.^15^, ^25^

### 2.5 Statistical Analysis

Pairwise Spearman correlation analysis was used to assess correlations between the 12 sex hormone measures. We assessed associations between levels of each sex hormone measure and mortality using multivariable Cox regression, providing hazard ratio (HR) with 95% confidence interval (CI), adjusted for covariates. The sex hormone measures were treated as categorical variables based on the cut‐off values of tertiles (three equal‐sized groups), except for progesterone, estradiol and androstenedione, where over one third of the values were below the limit of detection. Levels of these three sex hormones were instead categorized into three groups, that is, one group of values below the limit of detection and the other two groups of approximately equal sizes for the detectable values. Two Cox models were derived for each sex hormone, one basic model with adjustment for age and calendar year at diagnosis (both as continuous variables) and a full model with further adjustment for BMI (continuous), tobacco smoking (never or ever), physical activity (high/medium or low/inactive) and esophagectomy (yes or no). We also assessed possible exposure‐response relations by treating each sex hormone measure as a discrete variable (ie, values as 1, 2 or 3) in the Cox regression. Because of the differences in survival between patients who had undergone surgery and those who had not, we further stratified the analyses by esophagectomy for the main outcome, that is, disease‐specific mortality. An experienced biostatistician (FM) performed all analyses according to a predefined protocol, using the statistical software package SAS 9.4 for Windows (SAS Institute Inc., Cary, NC). All statistical tests were two sided.

## 3 RESULTS

### 3.1 Patients

Among all 244 male EAC patients included in our study, 89 (36.5%) underwent curatively intended surgery. The mean age at blood donation was 42.2 (SD ± 7.2) years, the mean age at EAC diagnosis was 66.5 (SD ± 8.5) years, and the mean duration from blood donation to EAC diagnosis was 24.4 (SD ± 8.5) years. Baseline characteristics of the patients are shown in Table 1.

**TABLE 1:** Characteristics of participants, presented as number (%)

Characteristics	Total	Surgical treatment	
		No	Yes
Total	244 (100)	155 (63.5)	89 (36.5)
Age at diagnosis, years			
<60	49 (20.0)	28 (18.1)	21 (23.6)
60 to 69	118 (48.4)	73 (46.5)	46 (51.7)
≥70	77 (31.6)	55 (35.5)	22 (24.7)
Mean *±* SD	66.5 ± 8.5	67.5 ± 9.2	64.8 ± 7.0
Year of diagnosis			
1985 to 1994	12 (4.9)	5 (3.2)	7 (7.9)
1995 to 2004	63 (25.8)	40 (25.8)	23 (25.8)
2005 to 2015	169 (69.3)	110 (71.0)	59 (66.3)
Tobacco smoking			
Never	39 (16.0)	20 (12.9)	19 (21.4)
Ever	184 (75.4)	122 (78.7)	62 (69.7)
Missing	21 (8.6)	13 (8.4)	8 (9.0)
Body mass index			
<25	84 (34.4)	50 (32.3)	34 (38.2)
25 to 29.9	113 (46.3)	74 (47.7)	39 (43.8)
≥30	28 (11.5)	20 (12.9)	8 (9.0)
Missing	19 (7.8)	11 (7.1)	8 (9.0)
Mean *±* SD	26.3 ± 3.3	26.3 ± 3.5	26.4 ± 3.0
Physical activity			
Inactive or low	167 (68.4)	105 (67.7)	62 (69.7)
Medium or high	57 (23.4)	37 (23.9)	20 (22.5)
Missing	20 (8.2)	13 (8.4)	7 (7.9)

A total of 212 (86.9%) patients died during the follow‐up, and 168 (68.9%) died from esophageal cancer until 31 December 2017. The median survival time was 0.8 (interquartile range 0.3‐1.5) years and the mean duration from blood donation to death was 25.4 (SD ± 8.8) years among the deceased patients.

### 3.2 Sex hormone levels

The distribution of serum levels of each sex hormone is presented in Supplementary Table 1. Pairwise Spearman correlation analysis provided the strongest coefficients for the pairings of testosterone and testosterone:estradiol ratio (0.62), testosterone and SHBG (0.61), FSH and luteinizing hormone (0.49), and testosterone and 17‐OH‐progesterone (0.49) (Supplementary Table 2).

### 3.3 Sex hormone levels and disease‐specific mortality

Patients with higher *SHBG* levels had decreased risk of disease‐specific mortality in EAC (fully adjusted HR 0.68, 95% CI 0.44‐1.07, highest vs lowest tertile) (Table 2). In analyses stratified by surgery, higher *SHBG* levels were associated with decreased disease‐specific mortality in nonoperated patients (adjusted HR 0.58, 95% CI 0.35‐0.96, highest vs lowest tertile), while no such decrease was found in operated patients (Table 3).

**TABLE 2:** Associations between prediagnostic levels and sex hormone measures and disease‐specific and all‐cause mortality in esophageal adenocarcinoma (N = 217)^a^

Sex hormone	Number of participants	Disease‐specific mortality		All‐cause mortality	
		Basic model HR (95% CI)^b^	Full model HR (95% CI)^c^	Basic model HR (95% CI)^b^	Full model HR (95% CI)^c^
Sex hormone‐binding globulin, nmol/L					
<33	72	1.00 (reference)	1.00 (reference)	1.00 (reference)	1.00 (reference)
33 to <47	65	0.82 (0.56‐1.22)	0.73 (0.47‐1.14)	0.76 (0.53‐1.08)	0.66 (0.45‐0.98)
≥47	80	0.74 (0.50‐1.11)	0.68 (0.44‐1.07)	0.75 (0.52‐1.06)	0.72 (0.49‐1.06)
Discrete		0.86 (0.71‐1.05)	0.83 (0.67‐1.04)	0.86 (0.72‐1.03)	0.86 (0.70‐1.05)
Dehydroepiandrosterone sulfate, μmol/L					
<5.5	72	1.00 (reference)	1.00 (reference)	1.00 (reference)	1.00 (reference)
5.5 to <7.8	69	1.23 (0.82‐1.83)	1.00 (0.67‐1.50)	1.14 (0.80‐1.64)	0.98 (0.68‐1.42)
≥7.8	76	1.09 (0.73‐1.64)	0.92 (0.61‐1.39)	1.05 (0.73‐1.50)	0.92 (0.63‐1.33)
Discrete		1.04 (0.86‐1.27)	0.96 (0.78‐1.18)	1.02 (0.86‐1.22)	0.96 (0.80‐1.15)
Follicle‐stimulating hormone, IU/L					
<3.2	65	1.00 (reference)	1.00 (reference)	1.00 (reference)	1.00 (reference)
3.2 to <4.8	78	1.45 (0.96‐2.18)	1.35 (0.89–2.05)	1.21 (0.85‐1.73)	1.19 (0.83‐1.70)
≥4.8	74	1.65 (1.10‐2.48)	1.61 (1.06‐2.43)	1.37 (0.96‐1.96)	1.35 (0.94‐1.94)
Discrete		1.28 (1.05‐1.56)	1.26 (1.03‐1.55)	1.17 (0.98‐1.40)	1.16 (0.97‐1.39)
Luteinizing hormone, IU/L					
<3.7	68	1.00 (reference)	1.00 (reference)	1.00 (reference)	1.00 (reference)
3.7 to <5.6	72	1.33 (0.89‐1.99)	1.27 (0.84‐1.90)	1.20 (0.84‐1.71)	1.19 (0.83‐1.71)
≥5.6	77	1.43 (0.95‐2.16)	1.38 (0.90‐2.10)	1.33 (0.92‐1.91)	1.36 (0.93‐1.98)
Discrete		1.19 (0.87‐1.45)	1.17 (0.95‐1.44)	1.15 (0.96‐1.38)	1.17 (0.97‐1.40)
Prolactin, mIU/L					
<107	71	1.00 (reference)	1.00 (reference)	1.00 (reference)	1.00 (reference)
107 to <170	78	1.13 (0.76‐1.67)	1.45 (0.97‐2.16)	1.08 (0.77‐1.53)	1.37 (0.96‐1.94)
≥170	68	0.78 (0.52‐1.17)	0.89 (0.59‐1.34)	0.65 (0.45–0.94)	0.74 (0.51–1.09)
Discrete		0.89 (0.73‐1.08)	0.96 (0.79‐1.17)	0.82 (0.69‐0.97)	0.89 (0.75‐1.07)
Testosterone, nmol/L					
<12.6	74	1.00 (reference)	1.00 (reference)	1.00 (reference)	1.00 (reference)
12.6 to <18.2	74	1.15 (0.78‐1.72)	0.89 (0.59‐1.32)	1.09 (0.77‐1.56)	0.87 (0.61‐1.25)
≥18.2	69	1.00 (0.67‐1.49)	0.88 (0.58‐1.33)	0.94 (0.66‐1.36)	0.89 (0.61‐1.30)
Discrete		0.99 (0.82‐1.21)	0.94 (0.76‐1.16)	0.97 (0.81‐1.16)	0.94 (0.78‐1.14)
17‐OH progesterone, nmol/L					
<1.5	71	1.00 (reference)	1.00 (reference)	1.00 (reference)	1.00 (reference)
1.5 to <2.3	70	0.99 (0.66‐1.48)	0.75 (0.50‐1.14)	1.02 (0.71‐1.47)	0.81 (0.56‐1.18)
≥2.3	76	1.10 (0.74‐1.63)	0.79 (0.53‐1.20)	1.25 (0.88‐1.79)	0.95 (0.65‐1.37)
Discrete		1.05 (0.86‐1.28)	0.90 (0.73‐1.10)	1.12 (0.94‐1.35)	0.98 (0.81‐1.18)
Progesterone, nmol/L					
≤1.1	135	1.00 (reference)	1.00 (reference)	1.00 (reference)	1.00 (reference)
>1.1 to 1.7	41	1.10 (0.72‐1.68)	1.01 (0.66‐1.56)	1.05 (0.72‐1.54)	0.97 (0.66‐1.43)
>1.7	41	1.29 (0.85‐1.96)	0.94 (0.60‐1.47)	1.33 (0.92‐1.93)	0.98 (0.66‐1.46)
Discrete		1.13 (0.93‐1.39)	0.98 (0.79‐1.21)	1.14 (0.95‐1.37)	0.99 (0.81‐1.20)
Estradiol, nmol/L					
>0.07	68	1.00 (reference)	1.00 (reference)	1.00 (reference)	1.00 (reference)
0.07 to 0.08	86	1.14 (0.77‐1.69)	1.10 (0.73‐1.66)	1.21 (0.84‐1.72)	1.23 (0.85‐1.78)
>0.08	63	1.19 (0.78‐1.80)	1.10 (0.71‐1.69)	1.38 (0.95‐2.01)	1.39 (0.94‐2.05)
Discrete		1.09 (0.89‐1.34)	1.05 (0.84‐1.30)	1.18 (0.98‐1.42)	1.18 (0.97‐1.43)
Androstenedione, nmol/L					
<2.7	78	1.00 (reference)	1.00 (reference)	1.00 (reference)	1.00 (reference)
2.7 to 4.3	68	1.10 (0.74‐1.62)	0.85 (0.57‐1.27)	1.21 (0.85‐1.72)	0.95 (0.66‐1.37)
>4.3	71	1.12 (0.76‐1.65)	1.10 (0.73‐1.65)	1.27 (0.90‐1.79)	1.29 (0.89‐1.87)
Discrete		1.06 (0.87‐1.28)	1.04 (0.84‐1.29)	1.13 (0.95‐1.34)	1.14 (0.94‐1.37)
Testosterone:estradiol ratio					
<184	70	1.00 (reference)	1.00 (reference)	1.00 (reference)	1.00 (reference)
184 to <254	73	1.17 (0.79‐1.72)	0.87 (0.58‐1.32)	1.10 (0.78‐1.56)	0.85 (0.59‐1.23)
≥254	74	1.08 (0.72‐1.62)	0.94 (0.62‐1.44)	0.95 (0.66–1.36)	0.83 (0.57‐1.21)
Discrete		1.04 (0.85‐1.27)	0.97 (0.79‐1.20)	0.97 (0.82‐1.16)	0.91 (0.76‐1.10)
Free testosterone index					
<3.7	75	1.00 (reference)	1.00 (reference)	1.00 (reference)	1.00 (reference)
3.7 to <4.6	72	1.14 (0.77‐1.68)	1.06 (0.71‐1.57)	1.22 (0.86‐1.75)	1.19 (0.83‐1.71)
≥4.6	70	1.06 (0.72‐1.57)	0.94 (0.63‐1.43)	1.19 (0.84‐1.69)	1.10 (0.76‐1.59)
Discrete		1.03 (0.85‐1.25)	0.97 (0.79‐1.19)	1.09 (0.92‐1.29)	1.05 (0.87‐1.26)

^a^ Among 244 patients, 27 patients were excluded from the analyses because of missing values of sex hormone measures, body mass index, tobacco smoking or physical activity.

^b^ Hazard ratio with 95% confidence interval, adjusted for age and calendar year at diagnosis (both as continuous variables).

^c^ Hazard ratio with 95% confidence interval, adjusted for age (continuous), calendar year at diagnosis (continuous), body mass index (continuous), tobacco smoking (never or ever), physical activity (high/medium, or low/inactive) and surgical treatment (yes or no).

**TABLE 3:** Associations between prediagnostic sex hormone levels and disease‐specific mortality in esophageal adenocarcinoma, stratified by surgical treatment (N = 217)^a^

Sex hormon	No surgical treatment		Surgical treatment	
	Number	HR (95% CI)^b^	Number	HR (95% CI)^b^
Sex‐hormone binding globulin, nmol/L				
<33	49	1.00 (reference)	23	1.00 (reference)
33 to <47	48	0.60 (0.36‐0.98)	24	1.42 (0.57‐3.53)
≥47	43	0.58 (0.35–0.96)	30	1.12 (0.46‐2.74)
Discrete		0.77 (0.59‐1.00)		1.05 (0.69‐1.60)
Dehydroepiandrosterone sulfate, μmol/L				
<5.5	44	1.00 (reference)	28	1.00 (reference)
5.5 to <7.8	46	1.01 (0.64‐1.61)	23	0.95 (0.41‐2.2)
≥7.8	50	0.91 (0.57‐1.46)	26	0.95 (0.41‐2.21)
Discrete		0.95 (0.76‐1.20)		0.98 (0.64‐1.49)
Follicle‐stimulating hormone, IU/L				
<3.2	42	1.00 (reference)	30	1.00 (reference)
3.2 to <4.8	48	1.49 (0.92–2.40)	23	0.96 (0.40–2.32)
≥4.8	50	1.64 (1.02–2.66)	24	1.56 (0.69–3.52)
Discret		1.27 (1.00‐1.60)		1.25 (0.82‐1.91)
Luteinizing hormone, IU/L				
<3.7	40	1.00 (reference)	28	1.00 (reference)
3.7 to <5.6	53	1.35 (0.84‐2.15)	23	1.03 (0.44‐2.39)
≥5.6	47	1.43 (0.87‐2.34)	26	1.24 (0.53‐2.88)
Discrete		1.19 (0.93‐1.51)		1.11 (0.72‐1.70)
Prolactin, mIU/L				
<107	49	1.00 (reference)	22	1.00 (reference)
107 to <170	45	1.43 (0.90‐2.25)	28	1.47 (0.62‐3.53)
≥ 170	46	0.92 (0.58‐1.45)	27	0.80 (0.32‐1.99)
Discrete		0.98 (0.79‐1.22)		0.89 (0.58‐1.35)
Testosterone, nmol/L				
<12.6	44	1.00 (reference)	26	1.00 (reference)
12.6 to <18.2	50	0.73 (0.47‐1.15)	23	1.76 (0.72‐4.30)
≥ 18.2	46	0.73 (0.46‐1.19)	28	1.44 (0.61‐3.41)
Discrete		0.86 (0.67‐1.10)		1.18 (0.78‐1.77)
17‐OH progesterone, nmol/L				
<1.5	42	1.00 (reference)	29	1.00 (reference)
1.5 to <2.3	45	0.63 (0.40‐1.00)	25	1.33 (0.56‐3.15)
≥2.3	53	0.67 (0.42‐1.06)	23	1.38 (0.59‐3.25)
Discrete		0.82 (0.65‐1.04)		1.18 (0.77‐1.79)
Progesterone, nmol/L				
≤1.1	83	1.00 (reference)	52	1.00 (reference)
>1.1 to 1.7	28	1.02 (0.63‐1.65)	13	0.95 (0.36‐2.52)
>1.7	29	0.89 (0.54‐1.47)	12	1.18 (0.45‐3.12)
Discrete		0.95 (0.75‐1.21)		1.06 (0.67‐1.70)
Estradiol, nmol/L				
<0.07	41	1.00 (reference)	27	1.00 (reference)
0.07 to 0.08	56	1.04 (0.65‐1.65)	30	1.23 (0.52‐2.90)
>0.08	43	0.94 (0.57‐1.55)	20	1.69 (0.72‐3.93)
Discrete		0.97 (0.75‐1.24)		1.30 (0.85‐2.00)
Androstenedione, nmol/L				
<2.7	48	1.00 (reference)	30	1.00 (reference)
2.7 to 4.3	47	0.75 (0.48‐1.18)	21	1.37 (0.59‐3.18)
>4.	45	1.10 (0.69‐1.75)	26	1.08 (0.47‐2.49)
Discrete		1.04 (0.82‐1.33)		1.04 (0.70‐1.56)
Testosterone:estradiol ratio				
<184	45	1.00 (reference)	27	1.00 (reference)
184 to <254	50	0.74 (0.46‐1.18)	22	1.57 (0.66‐3.76)
≥254	45	0.87 (0.54‐1.41)	28	1.12 (0.49‐2.56)
Discrete		0.94 (0.73‐1.21)		1.05 (0.71‐1.56)
Free testosterone index				
<3.7	42	1.00 (reference)	30	1.00 (reference)
3.7 to <4.6	47	0.98 (0.62‐1.55)	25	1.41 (0.63‐3.12)
≥4.6	51	1.00 (0.62‐1.61)	22	0.78 (0.32‐1.88)
Discrete		0.99 (0.78‐1.26)		0.91 (0.61‐1.37)

^a^ Among 244 patients, 27 patients were excluded from the analyses because of missing values of sex hormone measures, body mass index, tobacco smoking or physical activity.

^b^ Hazard ratio with 95% confidence interval, adjusted for age (continuous), calendar year at diagnosis (continuous), body mass index (continuous), tobacco smoking (never or ever) and physical activity (high/medium, or low/inactive).

Patients with higher *FSH* levels had increased disease‐specific mortality in an exposure‐response pattern (Table 2). Using the lowest tertile of *FSH* levels as the reference, the fully adjusted HRs for the middle and highest tertiles were 1.35 (95% CI 0.89‐2.05) and 1.61 (95% CI 1.06‐2.43), respectively. In stratified analyses by surgery, higher *FSH* levels were associated with increased disease‐specific mortality in patients who had not undergone surgery; adjusted HRs for the middle and highest were 1.49 (95% CI 0.92‐2.40) and 1.64 (95% CI 1.02‐2.66), respectively (Table 3). In the operated patients, the adjusted HRs for the middle and highest tertiles were 0.96 (95% CI 0.40‐2.32) and 1.56 (95% CI 0.69‐3.52), respectively (Table 3).

Higher luteinizing hormone levels were associated with increased point estimates of disease‐specific mortality (adjusted HR 1.38, 95% CI 0.90‐2.10, highest vs lowest tertile), but the risk estimates were not statistically significant (Tables 2 and 3).

No clear associations were observed between serum levels of dehydroepiandrosterone sulfate, prolactin, testosterone, 17‐OH‐progesterone, progesterone, estradiol, androstenedione, testosterone:estradiol ratio, or free testosterone index, and the risk of disease‐specific mortality in EAC patients (Tables 2 and 3).

### 3.4 Sex hormone levels and all‐cause mortality

Associations between levels of each sex hormone and all‐cause mortality are shown in Table 2. Higher *SHBG* levels were associated with decreased all‐cause mortality in EAC patients (fully adjusted HR 0.72, 95% CI 0.49‐1.06, highest vs lowest tertile). Associations between higher *FSH* levels and increased all‐cause mortality were indicated, but the risk estimates were not statistically significant (fully adjusted HR 1.35, 95% CI 0.94‐1.94, highest vs lowest tertile). Higher *prolactin* levels were associated with decreased all‐cause mortality in the basic model (HR 0.65, 95% CI 0.45‐0.94, highest vs lowest tertile), but the association was attenuated in the fully adjusted model (HR 0.74, 95% CI 0.51‐1.09). No clear associations were observed for the other nine sex hormone measures with the risk of all‐cause mortality.

## 4 DISCUSSION

Our study indicated an increased disease‐specific mortality with lower SHBG levels and higher FSH levels in male EAC patients without surgical treatment. No clear associations were observed for dehydroepiandrosterone sulfate, luteinizing hormone, prolactin, testosterone, 17‐OH‐progesterone, progesterone, estradiol, androstenedione, testosterone:estradiol ratio or free testosterone index.

To the best of our knowledge, our study is the first to assess associations between prediagnostic sex hormone levels and survival in EAC patients. The population‐based cohort design with complete follow‐up minimized selection bias. The sex hormone levels were measured prospectively, on average over 20 years before the EAC diagnosis, thus avoiding biased influence of the EAC or its treatment. The measurement in a single serum sample prevented longitudinal evaluation of sex hormone levels. However, prospective studies have suggested that one‐time blood measure captures long‐term exposure of sex hormone levels reasonably well and that there is a strong correlation between multiple blood measures over long periods of time.^26^, ^27^ We used the high‐accuracy mass spectrometry method for analyzing levels of testosterone, 17‐OH‐progesterone and androstenedione, while quantitation of the remaining hormones was conducted by immunoassays, which have lower sensitivity and specificity. Due to lack of data, we were not able to adjust or stratify the analyses for tumor stage. Instead, we used surgical treatment, which is closely related with tumor stage. Furthermore, because the sex hormone levels were measured in serum samples collected well before the EAC diagnosis, they were unlikely to be associated with tumor stage at diagnosis. Therefore, any unmeasured confounding by tumor stage should be negligible. Finally, chance errors could have occurred because of multiple testing and limited statistical power, particularly in the stratified analyses in the operated patients.

The observed associations between higher FSH levels and an increased disease‐specific mortality in EAC in our study are biologically plausible, and might thus not be a chance finding. Our recent Mendelian randomization analysis using data from international consortia of genome‐wide association studies suggested that higher genetically predicted levels of FSH increase the risk of EAC and Barrett's esophagus in both men and women.^28^ Other investigations have shown a dose‐response risk reduction of EAC associated with childbearing and breastfeeding, which strongly influence FSH levels.^29^ Additionally, the FSH receptor is highly expressed in the lower esophagus where EAC arises, possibly higher than in anywhere else in the human body.^30^ Promotion of tumor angiogenesis is a possible mechanism by which FSH may influence EAC survival, because the FSH receptor is selectively expressed on the endothelial surface of the blood vessels in several tumors.^31^

Our study also suggested a decreased disease‐specific mortality associated with higher SHBG levels in nonoperated male patients with EAC. Previous studies have consistently shown that higher SHBG levels decrease the risk of breast cancer in postmenopausal women.^32^, ^33^, ^34^ Anticancer properties of SHBG may be complex due to its pleiotropic actions^35^, ^36^ and could include activation of cyclic adenosine monophosphate (an important intracellular signal transduction pathway for cancer growth),^35^ increased apoptosis and regulation of cell growth.^37^, ^38^

Despite decades of efforts aimed at improving the treatment of EAC, the survival remains poor.^2^, ^4^ Whether sex hormone levels influence the survival in EAC requires more large and prospective investigations. Yet, if future research confirms the observed associations between FSH or SHBG levels and survival in EAC, evaluating potential therapeutic targets would be warranted. One such possibility could be blocking the FSH receptor, for example, in patients who receive palliative treatment or as adjuvant therapy in patients who undergo curatively intended treatment. Future studies may also investigate how FSH or SHBG levels change with the progression of esophageal adenocarcinoma to better inform potential therapeutic targets.

In summary, this population‐based and prospective cohort study indicates that higher prediagnostic endogenous levels of SHBG and lower levels of FSH improve the survival in male EAC patients. The other 10 examined sex hormone measures may not influence the survival in EAC.

## CONFLICT OF INTEREST

The authors declare no conflict of interest.

## ETHICS STATEMENT

The participants in the Janus cohort gave a broad consent, and the Regional Committee for Medical and Health Research Ethics in South‐Eastern Norway approved this specific study (reference number 2016/1114).

## Supporting information


**Appendix** **S1:** Supporting information

## Data Availability

The data that support the findings of this study are available from the corresponding author (SHX) upon reasonable request. The codes for the data analysis are archived by the biostatistician (FM).
