# Supplementary material for: Prediagnostic circulating levels of sex hormones and survival in esophageal adenocarcinoma
Source: Int J Cancer. 2020 Sep 22;148(4):905–13. doi: 10.1002/ijc.33285 (PMC7820945; doi:10.1002/ijc.33285)
Supplement: Supplementary file 1 — Appendix S1: Supporting information [file IJC-148-905-s001.pdf]

Pre-diagnostic Circulating Levels of Sex Hormones and Survival in Esophageal Adenocarcinoma

Shao-Hua Xie, Eivind Ness-Jensen, Hilde Langseth, Randi E. Gislefoss, Fredrik Mattsson, Jesper Lagergren

Supplementary Table 1. Distributions of circulating levels of sex hormone measures by surgical treatment

Supplementary Table 2. Pair-wise Spearman's correlation coefficients between circulating levels of sex hormone measures

Supplementary Table 1. Distributions of circulating levels of sex hormone measures by surgical treatment

Hormone	Surgical treatment	Mean	Standard deviation	Median	Lower quartile	Upper quartile	Minimum	Maximum
Sex hormone-binding globulin, nmol/L	No	39.8	14.5	37.0	28.0	49.5	14.0	84.0
	Yes	43.3	17.6	40.0	31.0	54.0	12.0	89.0
Dehydroepiandrosterone sulfate, µmol/L	No	7.0	3.4	7.1	4.6	8.6	0.3	21.2
	Yes	7.1	3.5	6.7	4.4	9.4	1.7	20.1
Follicle-stimulating hormone, IU/L	No	5.1	4.0	3.9	2.9	5.7	0.7	32.2
	Yes	4.2	2.2	3.9	2.7	4.9	0.7	13.5
Luteinizing hormone, IU/L	No	5.1	2.8	4.7	3.5	6.1	1.5	25.6
	Yes	4.7	1.8	4.6	3.2	6.1	1.5	10.2
Prolactin, mIU/L	No	150.7	91.4	140.5	80.5	187.5	28.0	627.0
	Yes	194.9	302.6	143.0	105.0	201.0	24.0	2690.0
Testosterone, nmol/L	No	16.2	6.0	15.3	11.8	19.6	1.9	38.7
	Yes	16.6	8.2	15.3	11.1	19.8	1.7	47.7
17-OH-progesterone, nmol/L	No	2.1	1	1.9	1.4	2.5	0.3	5.8
	Yes	2.1	1.4	1.8	1.2	2.4	0.1	8.9
Progesterone, nmol/L	No	1.4	0.5	1.1	1.1	1.7	1.1	3.6
	Yes	1.3	0.4	1.1	1.1	1.6	1.1	2.7
Estradiol, nmol/L	No	0.08	0.03	0.08	0.06	0.09	0.04	0.15
	Yes	0.07	0.02	0.07	0.06	0.09	0.04	0.13
Androstenedione, nmol/L	No	5.1	5.3	3.2	1.9	4.9	1.9	28.1
	Yes	5.5	6.5	3.1	1.9	5.8	1.9	33.7
Testosterone:estradiol ratio	No	226.0	93.8	218.6	168.7	268.8	44.8	669.4
	Yes	236.8	107.3	219.2	152.5	282.9	24.3	581.7
Free testosterone index	No	4.3	1.4	4.2	3.4	5.3	0.4	8.9
	Yes	4.0	1.4	3.9	3.0	4.7	0.4	8.9

Supplementary Table 2. Pair-wise Spearman's correlation coefficients between circulating levels of sex hormone measures

Hormone	DHEAS	FSH	LH	Prolactin	Testosterone	17-OHP	Progesterone	Estradiol	AE	T:E2 ratio	FTI
SHBG	0.013	0.114	0.271**	0.136*	0.613**	0.207**	0.057	0.212**	0.118	0.432**	-0.402**
DHEAS		-0.120	-0.006	0.014	0.056	-0.085	0.451**	0.076	0.135	-0.003	0.080
FSH			0.493**	0.015	0.113	-0.052	-0.062	0.147*	0.107	0.026	0.016
LH				0.102	0.240**	0.082	0.086	0.321**	0.192**	-0.018	-0.008
Prolactin					-0.017	-0.065	-0.106	-0.004	-0.104	-0.059	-0.164*
Testosterone						0.486**	0.067	0.395**	0.226**	0.623**	0.419**
17-OHP							0.105	0.119	0.250**	0.342**	0.356**
Progesterone								0.146	0.095	-0.027	-0.005
Estradiol									0.130	-0.399**	0.220**
AE										0.120	0.142*
T:E2 ratio											0.207**

* $P<0.05$; ** $P<0.01$

AE: androstenedione; DHEAS: dehydroepiandrosterone sulfate; FSH: follicle stimulating hormone; FTI: free testosterone index; LH: luteinizing hormone; 17-OHP: 17-OH-progesterone; SHBG: sex hormone-binding globulin; T:E2 ratio: testosterone:estradiol ratio
